# A Stroke Mimic Driven by Fear: Nonketotic Hyperglycemic Hemichorea in a Patient With Needle Phobia

**DOI:** 10.7759/cureus.108735

**Published:** 2026-05-12

**Authors:** Mohammed Haroun, Shreyashi Khanal, Sophio Kakabadze, Hala Abbas, Samantha Udarbe

**Affiliations:** 1 Internal Medicine, Ascension Saint Joseph, Chicago, USA; 2 Medicine, University of Illinois at Chicago, Chicago, USA

**Keywords:** diabetic hemi-chorea, needle phobia, non-ketotic hyperglycemia, striatopathy, stroke mimic

## Abstract

Nonketotic hyperglycemic hemichorea, also known as diabetic striatopathy, is a rare but reversible neurological complication of poorly controlled diabetes mellitus that can mimic acute cerebrovascular events.

We report a case of an elderly woman with poorly controlled type 2 diabetes mellitus (glycated hemoglobin (HbA1c) 12.6%) who presented with a 2-week history of progressive, involuntary movements involving the left upper and lower extremities. The movements were continuous, non-suppressible, and consistent with hemichorea. Laboratory evaluation revealed severe hyperglycemia (glucose >500 mg/dL), normal serum osmolality (295 mOsm/kg), and negative ketones, confirming a nonketotic state. Non-contrast computed tomography of the head demonstrated hyperdensity of the right caudate and putamen. Magnetic resonance imaging showed T1-weighted hyperintensity involving the right basal ganglia. The differential diagnosis included acute stroke and neurodegenerative etiologies; however, clinical and radiologic findings supported diabetic striatopathy. The patient was treated with intravenous fluids and insulin therapy, resulting in rapid glycemic control and significant improvement in symptoms without the need for neuroleptic agents. At the three-month follow-up, she demonstrated near-complete clinical resolution, with repeat imaging confirming near-complete resolution of prior abnormalities.

This case highlights nonketotic hyperglycemic hemichorea as a reversible stroke mimic and underscores the importance of early recognition and glycemic control. It also emphasizes how behavioral factors, such as needle phobia, can contribute to poor disease control and lead to significant neurological complications.

## Introduction

Chorea is characterized by involuntary, hyperkinetic movements usually involving the distal limbs, face, and trunk [1]. There are numerous etiologies for acquired chorea, including autoimmune, cerebrovascular, drug-induced, infectious, metabolic, neoplastic, and toxic causes [2]. One rare metabolic cause of chorea is hyperglycemia, which produces nonketotic hyperglycemic hemichorea--also referred to as diabetic striatopathy among several names in the literature, and is typically associated with longstanding, poorly controlled type 2 diabetes mellitus [3]. Unlike ketotic hyperglycemia, where ketone bodies serve as an alternative cerebral energy substrate, the absence of ketosis forces the brain to utilize gamma-aminobutyric acid (GABA) as a fuel source, depleting striatal inhibitory tone and precipitating hyperkinetic movement disorders [3]. Its prevalence is estimated at 1 in 100,000, and it has been reported more frequently in women of Asian descent [4]. The diagnosis is established by the presence of involuntary movements (chorea or ballism) and characteristic abnormalities in the basal ganglia on computed tomography (CT) and/or magnetic resonance imaging (MRI) [1]. The current standard of care is glycemic control, which typically improves chorea after several days to weeks [4]. We present a case of nonketotic hyperglycemic hemichorea precipitated by needle phobia-driven insulin noncompliance.

## Case presentation

An elderly woman with a history of poorly controlled type 2 diabetes mellitus, noncompliant with insulin therapy due to needle phobia, and with hypertension presented to the emergency department with a two-week history of progressive involuntary movements involving the left upper and lower extremities. The abnormal movements initially began in the left arm and subsequently involved the left leg over several days. She described the movements as continuous, involuntary, and without any preceding urge or ability to suppress them. She denied any prior history of similar symptoms.

The patient reported no associated headache, dizziness, speech difficulty, dysphagia, focal weakness, or sensory deficits. However, she endorsed mild gait impairment secondary to the abnormal movements. She also noted a subjective decline in vision in her left eye, for which she was awaiting outpatient ophthalmologic evaluation. One week prior to presentation, she was evaluated by her primary care physician for similar symptoms with concern for stroke and was initiated on additional antihyperglycemic therapy. Her medical history was notable for a remote cerebrovascular accident without residual deficits.

On examination, the patient was alert and oriented with normal speech and no cranial nerve deficits, including absence of facial droop. Motor examination revealed continuous, irregular, non-rhythmic involuntary movements involving the left upper extremity more than the lower extremity, consistent with hemichorea. Muscle tone was normal, and there was no clear focal weakness, although strength and coordination were difficult to fully assess due to the ongoing movements. Sensory examination was intact bilaterally, and she was able to ambulate with mild difficulty.

Laboratory evaluation was notable for severe hyperglycemia with a blood glucose level exceeding 500 mg/dL, HbA1c of 12.6%, and mild hyponatremia (serum sodium 131 mmol/L; corrected sodium 137 mmol/L). Serum osmolality was 295 mOsm/kg. Ketone levels were negative, supporting a nonketotic hyperglycemic state.

**Figure 1:**
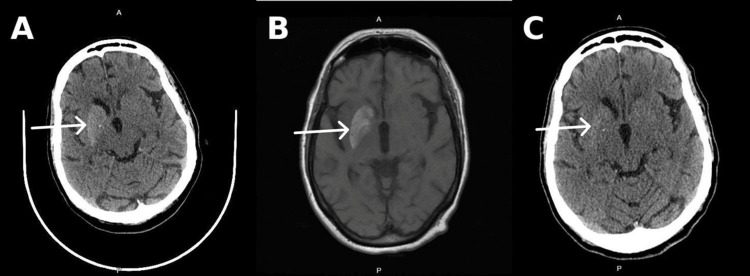
(A) Axial non-contrast computed tomography (CT) of the head at presentation demonstrating asymmetric hyperdensity of the right caudate nucleus and putamen compared to the contralateral side, without evidence of acute hemorrhage, mass effect, or midline shift (white arrow). (B) Axial T1-weighted magnetic resonance imaging (MRI) of the brain demonstrating striking hyperintensity involving the right caudate head and putamen, with relative sparing of the contralateral basal ganglia (white arrow), characteristic of nonketotic hyperglycemic hemichorea (diabetic striatopathy). (C) Axial non-contrast CT of the head at three-month follow-up demonstrating near-complete resolution of the previously observed right basal ganglia hyperdensity following sustained glycemic control with insulin therapy (white arrow).

Non-contrast CT of the head demonstrated asymmetric hyperdensity of the right caudate nucleus and putamen compared to the contralateral side, without evidence of acute hemorrhage, mass effect, or midline shift (Figure 1A). Magnetic resonance imaging of the brain demonstrated abnormal signal changes involving the right caudate head, putamen, and globus pallidus, characterized by T1-weighted hyperintensity, consistent with nonketotic hyperglycemic hemichorea (Figure 1B).

The differential diagnosis included acute cerebrovascular accident and neurodegenerative causes such as Huntington's disease; however, the subacute course, absence of focal neurologic deficits, characteristic imaging findings, and severe nonketotic hyperglycemia supported the diagnosis. The patient had no history of exposure to dopamine antagonists or other medications associated with secondary chorea.

The patient was treated with subcutaneous insulin, including both basal and short-acting regimens, resulting in gradual correction of hyperglycemia. Her blood glucose improved to 144 mg/dL within 24 hours. No neuroleptic or dopamine-depleting agents were required. She experienced significant clinical improvement, with approximately 60-70% reduction in abnormal movements, as stated by the patient, and complete resolution of lower extremity involvement.

The patient was discharged home on an insulin regimen using pen devices to address her needle phobia, with close outpatient follow-up arranged with neurology and endocrinology. At the three-month follow-up, her HbA1c was 7.3%, and she demonstrated near-complete resolution of abnormal movements. Repeat non-contrast CT of the head confirmed near-complete resolution of the previously observed right basal ganglia hyperdensity (Figure 1C).

## Discussion

This case highlights nonketotic hyperglycemic hemichorea (NHH) as a reversible stroke mimic, with a subacute presentation that can delay diagnosis. The absence of focal neurologic deficits, together with characteristic basal ganglia changes on imaging, was critical in distinguishing NHH from cerebrovascular and neurodegenerative causes. The diagnostic challenge in this case was further compounded by the patient's remote history of ischemic stroke, which, despite leaving no residual deficits and occurring in a different vascular territory, understandably heightened clinical concern for an acute recurrent cerebrovascular event. This history likely reinforced the initial stroke-first thinking, underscoring the importance of considering metabolic etiologies - particularly diabetic striatopathy - even in patients with prior cerebrovascular disease, when the clinical and laboratory picture supports an alternative diagnosis.

The precise pathophysiological mechanism underlying NHH remains incompletely understood, and several hypotheses have been proposed. The most widely accepted theory centers on metabolic dysfunction within the basal ganglia, specifically depletion of GABA. Under normal conditions, GABA serves as the primary inhibitory neurotransmitter of the indirect pathway of the basal ganglia circuit. In states of severe hyperglycemia without ketosis, the brain is thought to utilize GABA as an alternative energy substrate instead of ketone bodies, which are absent in the nonketotic state, leading to its depletion within the striatum. This depletion disinhibits the thalamocortical motor pathways, resulting in the hyperkinetic movements characteristic of chorea [3,5]. The reason ketosis appears protective against this phenomenon is thought to be that ketone bodies serve as an alternative fuel source, sparing GABA from metabolic consumption [4].

The characteristic neuroimaging findings of diabetic striatopathy further inform our understanding of its pathophysiology, though they too remain a subject of debate. On non-contrast CT, contralateral striatal hyperdensity is consistently observed, while MRI demonstrates T1-weighted hyperintensity in the affected basal ganglia. Several mechanisms have been proposed to explain these findings, including petechial hemorrhage, reactive gemistocytic astrogliosis, mineralization, and accumulation of paramagnetic substances, such as calcium, within the striatum [3]. Notably, the imaging findings typically resolve with sustained glycemic control, paralleling clinical improvement, as demonstrated in this case with near-complete radiologic resolution at three months. According to Chua et al., complete resolution of striatal hyperdensities on CT occurs at a median of 60 days, while complete resolution of T1-weighted MRI hyperintensities occurs at a median of 180 days, supporting the radiologic recovery observed in our patient at three-month follow-up [3].

The predilection of this condition for older women of Asian descent remains unexplained but may reflect differences in body composition, cerebrovascular reserve, or susceptibility to metabolic derangement within the basal ganglia [4].

A particularly notable aspect of this case is the role of needle phobia as a modifiable behavioral barrier to insulin adherence, ultimately contributing to prolonged uncontrolled hyperglycemia and its neurological sequelae. Fear of needles, known formally as trypanophobia, is estimated to affect approximately 0.2%-63.2% of adults, depending on the population and methodology used, and is a well-recognized but frequently underappreciated obstacle in diabetes management [6,7]. Among insulin-dependent patients, needle phobia has been associated with avoidance of self-injection, irregular dosing, and significantly poorer glycemic control [8]. Despite its prevalence and clinical impact, fear of injection is inconsistently screened for in routine diabetes care, leaving a substantial proportion of patients without targeted intervention [6].

In this patient, needle phobia resulted in chronic noncompliance with insulin therapy, reflected in her HbA1c of 12.6% at presentation. Importantly, the barrier was addressed through a practical, low-threshold intervention - transitioning from standard insulin syringes to pen devices, which are associated with reduced injection pain, greater dosing accuracy, and improved patient acceptability [9]. At the three-month follow-up, her HbA1c had improved dramatically to 7.3%, suggesting meaningful adherence following this intervention. This trajectory illustrates that identifying and addressing the specific nature of a patient's barrier, rather than simply reinforcing the need for compliance, can yield rapid and clinically significant results. While transitioning to insulin pen devices represented a practical first step in addressing this patient's needle phobia, it is important to acknowledge that both modalities still require needle use. Insulin pump therapy, particularly when integrated with continuous glucose monitoring (CGM) systems, represents a more comprehensive technological solution by substantially reducing the frequency of needle exposure and eliminating the need for repeated daily injections and fingerstick monitoring [10,11]. However, in this patient, insurance coverage limitations and financial constraints precluded access to these advanced diabetes technologies, highlighting a critical but frequently overlooked socioeconomic barrier to optimal diabetes management. This case underscores the need for broader insurance coverage and affordability initiatives for diabetes technology, particularly in patients with behavioral barriers, such as needle phobia, where such interventions may prevent serious and avoidable neurological complications.

This case argues for routine, structured screening for psychological barriers to insulin therapy, including needle phobia, as part of standard diabetes management. Tools such as the Diabetes Attitudes, Wishes and Needs (DAWN) study questionnaire and the Injection Technique Questionnaire (ITQ) exist for this purpose but remain underutilized in clinical practice [12,13]. A multidisciplinary approach incorporating diabetes educators, pharmacists, and mental health professionals, where appropriate, may further support patients in overcoming such barriers before they result in avoidable complications.

The patient's marked improvement with glycemic control alone, without the need for neuroleptic therapy, further emphasizes the importance of early recognition and appropriate management. Near-complete clinical and radiologic resolution at three months underscores the reversible nature of this condition.

This case has several strengths and limitations worthy of acknowledgment. The diagnosis was supported by both clinical presentation and characteristic neuroimaging findings on CT and MRI, with documented laboratory confirmation of nonketotic hyperglycemia, and near-complete clinical and radiologic resolution at three-month follow-up, providing strong evidence of reversibility. However, as a single case report, generalizability is inherently limited. Additionally, a formal psychological assessment of needle phobia severity was not performed, and adherence to insulin pen therapy was inferred from glycemic improvement rather than directly measured. Future studies incorporating structured screening tools for needle phobia and prospective assessment of diabetes technology interventions in this population would be valuable.

## Conclusions

Nonketotic hyperglycemic hemichorea is a rare but reversible neurological complication of poorly controlled diabetes mellitus that can closely mimic acute cerebrovascular events. Early recognition and prompt glycemic control remain the cornerstone of management, with the potential for significant clinical and radiologic recovery without neuroleptic therapy. This case uniquely illustrates how needle phobia can silently precipitate life-altering neurological complications through insulin nonadherence, highlighting the importance of routine screening for psychological barriers to insulin therapy. When behavioral barriers are identified, clinicians should explore the full spectrum of available interventions, from insulin pen devices to advanced diabetes technologies, such as insulin pump therapy and continuous glucose monitoring, while remaining mindful of the socioeconomic constraints that may limit access to optimal care.
